# Supplementary material for: Assessment of preventive behavior and associated factors towards COVID-19 in Qellam Wallaga Zone, Oromia, Ethiopia: A community-based cross-sectional study
Source: PLoS One. 2021 Apr 30;16(4):e0251062. doi: 10.1371/journal.pone.0251062 (PMC8087041; doi:10.1371/journal.pone.0251062)
Supplement: S1 Table — (DOCX) [file pone.0251062.s001.docx]

S1 Table: Summary steps for the final model in determining predictors of preventive behavior towards COVID-19

| **Variables in the Equation** | | | | | | | | | |
| --- | --- | --- | --- | --- | --- | --- | --- | --- | --- |
|  | | B | S.E. | Wald | df | Sig. | Exp(B) | 95% C.I.for EXP(B) | |
|  |  |  |  |  |  |  |  | Lower | Upper |
| Step 1^a^ | q8(1) | 1.087 | .341 | 10.177 | 1 | .001 | 2.965 | 1.521 | 5.782 |
|  | Constant | -2.930 | .309 | 89.646 | 1 | .000 | .053 |  |  |
| Step 2^b^ | q8(1) | 1.076 | .342 | 9.881 | 1 | .002 | 2.932 | 1.499 | 5.733 |
|  | q12_4(1) | .828 | .294 | 7.911 | 1 | .005 | 2.288 | 1.285 | 4.073 |
|  | Constant | -3.101 | .320 | 93.670 | 1 | .000 | .045 |  |  |
| Step 3^c^ | Knowledgenew(1) | .775 | .294 | 6.968 | 1 | .008 | 2.170 | 1.221 | 3.858 |
|  | q8(1) | 1.173 | .347 | 11.441 | 1 | .001 | 3.233 | 1.638 | 6.380 |
|  | q12_4(1) | .814 | .297 | 7.512 | 1 | .006 | 2.257 | 1.261 | 4.040 |
|  | Constant | -3.362 | .344 | 95.720 | 1 | .000 | .035 |  |  |
| a. Variable(s) entered on step 1: q8. | | | | | | | | | |
| b. Variable(s) entered on step 2: q12_4. | | | | | | | | | |
| c. Variable(s) entered on step 3: Knowledgenew4. | | | | | | | | | |
