# Supplementary material for: Assessment of preventive behavior and associated factors towards COVID-19 in Qellam Wallaga Zone, Oromia, Ethiopia: A community-based cross-sectional study
Source: PLoS One. 2021 Apr 30;16(4):e0251062. doi: 10.1371/journal.pone.0251062 (PMC8087041; doi:10.1371/journal.pone.0251062)
Supplement: S1 Questionnaire — (DOCX) [file pone.0251062.s003.docx]

# Annex 1: English version of the study questionnaire

Code: ______________ District: _____________________________Ganda: _______________________

**Dambi Dollo University**

**College Of Medicine and Health Science**

**Questionnaire for assessment of preventive behavior for covid-19 and associated factors in Qellam Wallaga Zone, Oromia, 2020**

**Consent sheet**

**Instruction:** Data collectors should read the entire consent for the participants as it is!

**Dear respondent**: Greetings

Ensuring the prevention and control of covid-19 requires understanding existing problems and related behaviors in the community. In line with we proposed to survey preventive behavior for covid-19 and associated factors in Qellam Wallaga zone Oromia, 2020. While all households in Qellam Wallaga are eligible, we interview only a sample of 634 households and you are randomly chosen to participate in this study.

The purpose of this study is to assess preventive behavior for COVID-19 and associated factors in Qellam Wallaga zone to generate baseline data that will be used to design appropriate interventions to respond to the outbreak. There is no way in which participating in this study can cause harm to the participants. No individual answer will be reported in this study and also it is your full right to refuse to answer some or all of the questions. The study will involve various socio-demographic, perception/opinion, knowledge ad practice questions related to COVID-19.

Your participation will have a big share to effectively attain the goal of this study. Here are a survey questions for you which will take a few minutes to complete. There is no need to give your name to the interviewer.

Do you mind participating in this study, please?

1. Yes, I want to participate in the study. (Please go to the next page)
2. No, I don't want to participate in the study.

- Thank you very much for your cooperation‼

| **Part I: socio-demographic characteristics** | | | |
| --- | --- | --- | --- |
|  | Age (in year) | | ----- |
|  | Sex | | 1. Male 2. Female |
|  | Educational status | | 1. Has no formal education 2. Literate (grade completed) ________ |
|  | Occupational status | | 1. Farmer 2. Gov’t employee 3. Merchant 4. Student 5. Daily laborer 6. Other, mention________________ |
|  | Religion | | 1. Orthodox 2. Protestant 3. Muslim 4. Others ________________ |
|  | Ethnicity | | 1. Oromo 2. Amhara 3. Gurage 4. Others_________________ |
|  | Marital status | | 1. Single 2. Married 3. Divorced 4. Widowed |
|  | Place of residence | | 1. Urban 2. Rural |
| **Part II: Access to information and its sources** | | | |
|  | Where did you hear about the new coronavirus from? What channels or sources? | ***Circle all channels that the respondents heard about the disease from:***   1. Heard about the disease from radio 2. Heard about the disease from TV 3. Heard about the disease from social media 4. Heard about the disease from a health unit/health care worker 5. Heard about the disease from family members 6. Heard about the disease from friends 7. Heard about the disease from community leaders 8. Heard about the disease from religious leaders 9. Heard about the disease from traditional healers 10. Heard about the disease from someone in the community | |
|  | What kind of information have you received about COVID-19? | ***Circle all sentence that the respondents replied “Yes”***   1. Heard about how to protect yourself from the disease? 2. Heard about the Symptoms of the new coronavirus disease? 3. Heard about how it is transmitted? 4. Heard about what to do if you have the symptoms? 5. Heard about risks and complications? | |
|  | Which channel/who do you most trust to receive information related to coronavirus? (one or more options) | ***Circle all the information channels the respondents trust to receive information from:***   1. Most trust information from phone/Ethiotelecom 2. Most trust information from radio 3. Most trust information from TV 4. Most trust information from social Media 5. Most trust information from the health unit/health care worker 6. Most trust information from family members 7. Most trust information from friends 8. Most trust information from community leaders 9. Most trust information from religious leaders 10. Most trust information from traditional healers 11. Most trust information from any person from the community | |
| **Part III: Knowledge towards COVID-19** | | | |
|  | How does the coronavirus spread? | ***Respond to all the questions by giving your answer from the given alternatives:***   1. Blood transfusion: 1. Yes 2. No 3. I don’t know 2. Droplets from infected people: 1. Yes 2. No 3. I don’t know 3. Direct contact with infected people: 1. Yes 2. No 3. I don’t know 4. Touching contaminated objects/surfaces: 1. Yes 2. No 3. I don’t know 5. Sexual intercourse contact: 1. Yes 2. No 3. I don’t know 6. Mosquito bites: 1. Yes 2. No 3. I don’t know 7. Drinking unclean water: 1. Yes 2. No 3. I don’t know | |
|  | There is no specific treatment or vaccine available for novel coronavirus as yet. | 1. Yes 2. No 3. Don´t know | |
|  | The novel coronavirus affects older people only | 1. Yes 2. No 3. Don´t know | |
|  | A person infected  with coronavirus  can completely recover  and be no more  infectious | 1. Yes 2. No 3. .Don´t know | |
|  | What are the symptoms of COVID-19 | ***Respond to all the questions by giving your answer from the given alternatives:***   1. Fever: 1. Yes 2. No 3. I don’t know 2. Dry cough: 1. Yes 2. No 3. I don’t know 3. Shortness of breath: 1. Yes 2. No 3. I don’t know 4. Muscle pain: 1. Yes 2. No 3. I don’t know 5. Headache: 1. Yes 2. No 3. I don’t know 6. Diarrhea: 1. Yes 2. No 3. I don’t know 7. Sore throat: 1. Yes 2. No 3. I don’t know | |
|  | Do you know how to prevent coronavirus? (One or more options) | ***Respond to all the questions giving your answer from the given alternatives:***   1. Sleep under the mosquito net: 1. Yes 2. No 3. I don’t know 2. Wash your hands regularly using alcohol or soap and water: 1. Yes 2. No 3. I don’t know 3. Cover your mouth and nose when coughing or sneezing: 1. Yes 2. No 3. I don’t know 4. Avoid close contact with anyone who has a fever and cough: 1. Yes 2. No 3. I don’t know 5. Eliminate standing water: 1. Yes 2. No 3. I don’t know 6. Cook meat and eggs well: 1. Yes 2. No 3. I don’t know 7. Avoid hand contact: 1. Yes 2. No 3. I don’t know | |
| **Part IV: Perceptions about COVID-19** | | | |
|  | How dangerous do you think the new coronavirus? | ***Choose the best answer that matches your belief about the disease***:   1. Very dangerous 2. More or less dangerous 3. Is not dangerous 4. Don't know | |
|  | What do you do if you may have contact with a suspected case with COVID-19? | 1. Go to the health facility for a checkup 2. Self-quarantine at my home 3. Call on 8335 (local free line for COVID-19) 4. Stay home till symptom develops 5. Other ___________________________ | |
|  | Do you think you are likely to become sick with the new coronavirus? | 1. Yes 2. No 3. Don´t know | |
|  | If yes to the above question, how do you rate your risk of contracting the new coronavirus? | 1. Very high 2. High 3. Moderate 4. Low 5. Very low/Rare | |
|  | Do you think you can protect yourself from the new coronavirus? | 1. Yes 2. No 3. Don´t know | |
|  | Do you think that there are treatments that can cure COVID-19? | 1. Yes 2. No 3. Don´t know | |
|  | Do you think that there are home remedies that can protect against coronavirus? | 1. Yes 2. No 3. Don´t know | |
|  | If yes to the above question, which home remedies do you think can protect you from the disease? | 1. Eating garlic: 1. Yes 2. No 2. Eating zinger: 1. Yes 2. No 3. Eating afagn (local food with excess spice): 1. Yes 2. No 4. Areke (local alcohol) : 1. Yes 2. No 5. Heto (traditional medicine for intestinal worms): 1. Yes 2. No | |
| **Part V: Preventive behaviors** | | | |
|  | How frequently have you applied the following corona preventive measures in the past two days? | ***Rate all the preventive statements that you have taken depending on the frequency of your action as:*** *1=Never, 2=Rarely, 3=Sometimes, 4=Most of the time, and 5=Always*   1. Wash your hand regularly with plenty of soap and water. ____ 2. Keep an alcohol-based sanitizer ready for times when soap and water are not available.____ 3. Don’t touch your eyes, mouth, or nose with unclean hands. 4. Use a tissue and throw it away immediately, or sneeze in the inner side of your elbow.____ 5. Keep your distance of at least 2 meters from anyone ____ 6. Cook meat and eggs well before eating 7. Follow no-touch greeting for no germs contracted.____ 8. Don’t travel or visit crowded places.____ | |

Name of data Collector: ___________________________ Sign. _________________ Date: __________

Name of supervisor: _____________________________ Sign. ___________________Date: _________
