# Supplementary material for: Assessment of preventive behavior and associated factors towards COVID-19 in Qellam Wallaga Zone, Oromia, Ethiopia: A community-based cross-sectional study
Source: PLoS One. 2021 Apr 30;16(4):e0251062. doi: 10.1371/journal.pone.0251062 (PMC8087041; doi:10.1371/journal.pone.0251062)
Supplement: S2 Questionnaire — (DOCX) [file pone.0251062.s004.docx]

# Annex II: Questionnaire Afan Oromo version

**Koodii____________ Aanaa_________ Ganda_______________**

**Yunivarsiitii Dambii Dollo**

**Kolleejjii Meedikaalaafi Saayinsii Fayyaa**

**Qorannaa COVID-19 irrattiti amala of”eeggannaa fii waantota kanaan hidhata qaban ilaalchisee godina Qeellam Wallagaa, naanoo Oromiyaa keessatti bara 2020 hojjetamu**

**Waliigaltee**

**Ibsa:** namni daataa funaanu waliigaltee kana akka jirutti hirmaattota hundaaf dubisuu qaba

**Kabajamoo hirmaataa keenya-(nagaa gaafadhu)**

Dhukkuba koronaa ittisuufii to’achuuf rakkoo karaa amaloota namaa jiraachuu malanifi kanaan walitti hidhata qaban beekuun barbaachisaa ta’a. kanuma irraa kan ka’e, amala of”eeggannaa fii waantota kanaan hidhata qaban ilaalchisee naannoo Oromiyaa godina Qeellam Wallagaa keessatti qorannoo gaggeessuf propozaaliin kun qophaa’ee jira. Manneen godina Qellam Wallagaa keessa jiran hundi carraan hirmaachuu isaanii qixa ta’ee osoo jiru manneen 634 qofatu barbaachisa waan ta’eef, isaan kana immo carra walqixa ta’een bifa lotooriitiin filachaa jirra. Isininis bifuma kanaan gaffii fii deebii kanaaf filatamtani jirtu.

Kaayyoon qorannoo kanaa amalaa fi waantota amala namaatiin walqabatanii ittisa dhukkuba koronaa irratti dhiibbaa geessisan adda baasuun odeeffanno bu’uuraa maddisiisuun dalagaa ittisaafi to’annaa irratti qophii taasisuudha. Hirmaattota qorannoo kana irratti hirmaatan karaa kamiinuu rakkon isaan irratti qaqabu hinjiru. Deebiin nama dhuunfaa gabaafamu hin jiru. Qorannoo kana irratti hirmaachuu dhiisuun ykn gaaffii hinfeene deebiisuu dhiisuu mirga keessani. Maqaa keessan ibsuun hinbarbaachisu. Qorannoon kun gaffiiwwan bu’uuraa kan akka hawaasummaa, ilaalcha, beekumsaa fii raawwii ittisa koronaatiin walqabatu of keessaa qaba.

Kabajamoo hirmaataa keenya, hirmaannaan keessan qorannaa kana galmaan ga’uuf gumaacha guddaa godha. Yoo eyyama keessan itti aansee gaaffii fii debiin keenya itti fufna. Gaaffii fii deebii itti fufuu dandeenya?

1. *Eeyyen, ni dandeenya!*
2. *Lakki, hindandeenyu! Galatoomaa!*

| **Part I: socio-demographic characteristics** | | |
| --- | --- | --- |
|  | Umurii (waggaa guutuun) | ----- |
|  | Saala | 1. Dhiira 2. Dhalaa |
|  | Sadarkaa barnootaa | 1. Kan hin baranne 2. Kan barate/tte (kutaa meeqaa) ________ |
|  | Gosa hojii | 1. Qonnaan bulaa 2. Hojjetaa/ttuu mootummaa 3. Daldalaa/ttuu 4. Barataa/ttuu 5. Hojii humnaan bulaa/ttuu 6. Kan biraa, ibsi________________ |
|  | Amantii | 1. Ortoodoksii 2. Pirotestaantii 3. Musiliima 4. Kan biraa, ibsi ________________ |
|  | Sabummaa | 1. Oromoo 2. Amhaaraa 3. Guraagee 4. kan biraa, ibsi_________________ |
|  | Haala gaa’elaa | 1. Kan hin ffune/hin heerumne 2. Kan fuudhe/heerumte 3. Kan hike/hiikte 4. Kan irraa du’e/jalaa duute |
|  | Iddoo jireenyaa | 1. Magaalaa 2. Baadiyyaa |
| 1. **Kutaa II: Odeeffannoo koronaa fi madda isaa** | | |
|  | Waa’ee COVID-19 eessaa ykn maal irraa dhageesse? | Kan sirrii ta’e hunda itti mari:   1. Raadiyoo 2. TV 3. Miidiyaalee hawaasaa 4. Dhaabbata fayyaa/ogeessa fayyaa 5. Miseensa maatii 6. Hiriyaa 7. Dursaa hawaasaa irraa 8. Abboota amantii irraa 9. Ogeessota fayyaa kan aadaa irraa/Traditional healers 10. Nama kan biraa kan hawaasaa keessaa irraa 11. Kan biraa, ibsi______________________ |
|  | Waa’ee COVID-19 odeeffannoo akkamii qabda? | Kan sirrii ta’e hunda sororsi:   1. Akkan itti dhukkuba kana irraa ofi eegu dhagaheen jira 2. Mallattoolee dhukkuba kanaa nan dhagaheen jira 3. Karaa daddarba isaa dhagaheen jira 4. Yoon mallattoolee isaa ofi irratti arge waanan godhu dhagaheen jira 5. Saaxila ba’ummaa fi miidhaa isaa dhagaheen jira |
|  | Maddoota odeeffannoo waa’ee COVID-19 kana keessaa kamiin caalaatti amanta?) | Kan sirrii ta’e hunda soroorsi.   1. Kan Bilbila 2. Kan Raadiyoo 3. Kan TV 4. Kan miidiyaalee hawaasaa 5. Kan dhaabbata fayyaa/ogeessa fayyaa 6. Kan miseensa maatii 7. Kan hiriyaa 8. Kan dursaa hawaasaa irraa 9. Kan abboota amantii irraa 10. Kan ogeessota fayyaa kan aadaa irraa/traditional healers 11. Kan nama biraa kan hawaasaa keessaa irraa |
|  | **Part III: beekumsa walii gala waa’ee COVID-19** | |
|  | Koroonaa vaayirasiin akkamitti tatamsa’a? | Kan sirrii ta’e hunda sororsi:   1. Karaa dhiiga waliif kennuu: 1. Eeyyen 2. Lakki3. Hinbeeku 2. Gorora/Droplets nama dhukkuba sana qabu irraa: 1. Eeyyen 2. Lakki3. Hinbeeku 3. Nama dhukkuba sana qabuun kallattiin wal tuttuquun: 1. Eeyyen 2. Lakki3. Hinbeeku 4. Wantoota virus sanaan faalame qaqqabachuun: 1. Eeyyen 2. Lakki3. Hinbeeku 5. Karaa wal quunnamtii saalaa: 1. Eeyyen 2. Lakki3. Hinbeeku 6. Bookeen ciniinamuun: 1. Eeyyen 2. Lakki3. Hinbeeku 7. Bishaan qulqulluu hin taane dhuguun: 1. Eeyyen 2. Lakki3. Hinbeeku |
|  | Hanga yoonaatti COVID-19 qorichas ta’e talaallii qabaa? | 1. Eeyyee 2. Lakkii 3. Hin beeku |
|  | COVI-19 namoota dulloomoo qofa miidha/qaba | 1. Eeyyee 2. Lakkii 3. Hin beeku |
|  | Namni COVID-19 qabame tokko guutummaatti fayyee dhukkubichas daddabarsuu dhiisuu danda’aa? | 1. Eeyyee 2. Lakkii 3. Hin beeku |
|  | Mallattooleen COVID-19 maal fa’i? | Kan sirrii ta’e hunda sororsi:   1. Ho’ina qaamaa dabaluu: 1. Eeyyen 2. Lakki3. Hinbeeku 2. Qufaa gogaa: 1. Eeyyen 2. Lakki3. Hinbeeku 3. Hafuura kutuu/harganuu dadhabuu: 1. Eeyyen 2. Lakki3. Hinbeeku 4. Dhukkubbii maashaa/muscle pain : 1. Eeyyen 2. Lakki3. Hinbeeku 5. Mataa cabsaa/dhukkubbii/bowwoo: 1. Eeyyen 2. Lakki3. Hinbeeku 6. Garaa kaasaa: 1. Eeyyen 2. Lakki3. Hinbeeku 7. Dhukkubbii qoonqoo: 1. Eeyyen 2. Lakki3. Hinbeeku |
|  | Malootni ittiin COVID-19 ittisan maal fa’i? | Kan sirrii ta’e hunda sororsi:   1. Saaphana(agoobara) bookee ittisu jala rafuu: 1. Eeyyen 2. Lakki3. Hinbeeku 2. Harka yeroo hundaa/irra deddeebiin bishaanii fi saamuunaan dhiqachuu: 1. Eeyyen 2. Lakki3. Hinbeeku 3. Yeroo qufaatuu fi haxxiffattu afaanii fi funyaan haguuggachuu: 1. Eeyyen 2. Lakki3. Hinbeeku 4. Nama dhaqna gubaa fi qufaa qabutti dhiyaachuu dhiisuu: 1. Eeyyen 2. Lakki3. Hinbeeku 5. Bishaan kuufamee jiru dhabamsiisuun : 1. Eeyyen 2. Lakki3. Hinbeeku 6. Foonii fi hanqaaquu sirriitti bilcheessanii nyaachuu: 1. Eeyyen 2. Lakki3. Hinbeeku 7. Harka wal-qabuu dhiisuu: 1. Eeyyen 2. Lakki3. Hinbeeku |
|  | **Part IV: Perception about risk of COVID-19** | |
|  | Dhukkubni kuni hangam hammaataa sitti fakkaata? | Filannoo yaada kee siif ibsuu danda’u filadhu   1. Baay’ee hammaataa 2. Hammaataa natti fakkaata 3. Hammaataa miti (salphaa dha) 4. Hin beeku |
|  | Nama dhukkuba kanaan shakkame waliin yoo tuttuqqii/hariiroo qabaatte maal goota? | 1. Gara mana yaalaan deema 2. Mana koottin adda ofi baasa (kutaa qofaa keessatti) 3. 8335 irrattin bilbilee beeksisa 4. Hanga mallattoon isaa ana irratti mul’atutti mana koon tura 5. Kan biraa, ibsi_____________ |
|  | Dhukkubni kuni naqabuu danda’a jettee ni yaaddaa? | 1. Eeyyee 2. Lakkii 3. Hin beeku |
|  | Gaaffii 23ffaaf deebiin kee eeyyee yoo ta’e sadarkaan saaxilamummaa keetii hangami sitti fakkaata? | 1. Baay’ee oli aanaadha 2. Oli aanaadha 3. Giddu galeessa 4. Gadi aanaa dha 5. Baay’ee gadi aanaa dha |
|  | Dhukkuba kana irraa ofi eeguu nan danda’a jettee yaaddaa? | 1. Eeyyee 2. Lakki 3. Hin beeku |
|  | Dhukkuba kana qorich ammayyaa fayyisuu dandau jira jettee yaaddaa? | 1. Eeyyee 2. Lakki 3. Hin beeku |
|  | Qorichi aadaa dhukkuba kana nama irraa ittisu jira jettee yaaddaa? | 1. Eeyyee 2. Lakkii 3. Hin beeku |
|  | Gaaffii kana 27’f deebiin kee eeyyee yoo ta’e maal fa’i? | 1. Qullubbii: 1. Eeyyen 2. Lakki 2. Jibinbila: 1. Eeyyen 2. Lakki 3. Affaanyi/Ukkaamsaa: 1. Eeyyen 2. Lakki 4. Araqee: 1. Eeyyen 2. Lakki 5. Heexoo: 1. Eeyyen 2. Lakki |
| **Part V: Preventive behavior** | | |
|  | Maloota ittisa COVID-19 kanaa gadii atii fi maatiin kee guyyoota lamaan darban keessatti hangam hojii irra oolchitaniittu? | Akka kana gadiitti raawwii keerratti hundaa’uun sadrakaa kenniif; 1= tasa hojii irra hin oolchinee, 2= darbe darbee(rarely), 3= yeroo jiddugaleessa 4=yeroo baay’ee, 5= yeroo hundaa   1. Harka samuunaa fi bishaaniin irra deddeebiin dhiqachuu 2. Yeroo saamuunaa fi bishaan hin jirre alkoolii fayyadamuu 3. Harka hin qulqulloofneen afaan, funyaan fi ija qabachuu dhiisuu 4. Yeroo qufaanu ykn haxxiffannu sooftii ykn keessa ciqilee keenyaa fayyadamuu 5. Fageenya meetira lamaa namoota biroo irraa eeggachuu 6. Foonii fi hanqaaquu utuu hin nyaatin sirriitti bilcheessuu 7. Nama harka qabanii dubbisuu dhiisuu 8. Iddoo namootni hedduun jiran deemuu dhiisuu |

Maqaa nama raga funaanee:______________________ mallattoo______________ Guyyaa___________

Maqaa Toa’ataa ______________________________ Mallattoo _________________ Guyyaa ________
