# Supplementary material for: Assessment of preventive behavior and associated factors towards COVID-19 in Qellam Wallaga Zone, Oromia, Ethiopia: A community-based cross-sectional study
Source: PLoS One. 2021 Apr 30;16(4):e0251062. doi: 10.1371/journal.pone.0251062 (PMC8087041; doi:10.1371/journal.pone.0251062)
Supplement: Attachment — Submitted filename: Response to Reviewers.docx [file pone.0251062.s005.docx]

| Date: | April 10 2021 |
| --- | --- |
| To: | " PLOS ONE " plosone@plos.org |
| From: | " Birhanu Gutu" birhanugutu@yahoo.com |
| Subject: | 'Response to Reviewers' [PONE-D-20-28112] |

PONE-D-20-28112
Assessment of preventive behavior and associated factors towards COVID-19 in Qellam Wallaga Zone, Oromia, Ethiopia: A community based cross-sectional study

Dear PLOS ONE academic editor(s) and experts/reviewer(s)

Thank you for generous second time comments which helped digs for further scientific standards and procedures.

**Response to Reviewers and academic editor’s comments**

1. If the authors have adequately addressed your comments raised in a previous round of review and you feel that this manuscript is now acceptable for publication, you may indicate that here to bypass the “Comments to the Author” section, enter your conflict of interest statement in the “Confidential to Editor” section, and submit your "Accept" recommendation.

Reviewer #1: All comments have been addressed

- **Authors’ Response:** thank you for accepting the revision

2. Is the manuscript technically sounds, and do the data support the conclusions?

The manuscript must describe a technically sound piece of scientific research with data that supports the conclusions. Experiments must have been conducted rigorously, with appropriate controls, replication, and sample sizes. The conclusions must be drawn appropriately based on the data presented.

 Reviewer #1: Yes

- **Authors’ Response:** thank you for accepting the conclusion

3. Has the statistical analysis been performed appropriately and rigorously?

 Reviewer #1: I Don't Know

- **Authors’ Response:** the authors believe that the statistical analysis been performed appropriately and rigorously and fulfills all the necessary steps.

4. Have the authors made all data underlying the findings in their manuscript fully available?

The [PLOS Data policy](http://www.plosone.org/static/policies.action#sharing) requires authors to make all data underlying the findings described in their manuscript fully available without restriction, with rare exception (please refer to the Data Availability Statement in the manuscript PDF file). The data should be provided as part of the manuscript or its supporting information, or deposited to a public repository. For example, in addition to summary statistics, the data points behind means, medians and variance measures should be available. If there are restrictions on publicly sharing data—e.g. participant privacy or use of data from a third party—those must be specified.

Reviewer #1: Yes

- **Authors’ Response:** thank you for accepting and addressing the availability of the data

5. Is the manuscript presented in an intelligible fashion and written in standard English?

PLOS ONE does not copyedit accepted manuscripts, so the language in submitted articles must be clear, correct, and unambiguous. Any typographical or grammatical errors should be corrected at revision, so please note any specific errors here.

 Reviewer #1: Yes

- **Authors’ Response:** thank you for your clarification about the manuscript’s presentation in an intelligible fashion and written in standard English

6. Review Comments to the Author

Please use the space provided to explain your answers to the questions above. You may also include additional comments for the author, including concerns about dual publication, research ethics, or publication ethics. (Please upload your review as an attachment if it exceeds 20,000 characters)

 Reviewer #1: Thank you for accepting the recommendations and congratulation for your efforts.

First, about the statistical analysis, I wonder if in your original procedures you applied a binomial logit regression (to estimate odds ratios) instead of a binomial log (to estimate prevalence ratios). My initial suggestion according to your study design was to update your analysis in case if it is required.

Also, I suggest to detail if you applied weights for survey analysis as explained here: https://dev.stats.idre.ucla.edu/stata/faq/can-i-use-working-weights-for-survey-analyses-in-spss/

Additionally, even though your statistical analysis followed a predictive procedure, have you evaluate how other covariates could affect the associations that you found? For example, the association between residence-behavior and knowledge-behavior could depend or be confounded by age, education or occupation. In case any of those apply as confounders, the reported estimates should be adjusted for this set of variables. This would not require to add more rows to the table 5, but specifing it as a caption or note at the end.

Related to this last point, I suggest to evaluate the consistency of the term "predictor" (as in subtitle 4.5) if you already applied "associated factor". Predictive modelling involve different objectives and procedures. Review Shmueli, Galit. "To explain or to predict?." Statistical science 25.3 (2010): 289-310. (https://doi.org/10.1214/10-STS330) and Chen, Lingxiao. "Overview of clinical prediction models." Annals of translational medicine 8.4 (2020). (https://doi.org/10.1214/10-STS330).

Furthermore, I recommend to add a limitation paragraph in the discussion section. There you could detail how your results may be affected by the validity or biases of the scale that you applied to define the outcome (preventive behavior), including references of previous experiences, if it apply. Also about the sampling procedure or the absence of sampling weighting procedure.

Lastly, I recommend to fix some writing typos like in lines 193, 194 or 223 from the "clean" copy of the manuscript. At line 188, it should end as "log link function". At table 4, you could add N(%) at the heading as in previous tables. At table 5, change the number "1" with "Ref." to detail the category of reference.

- **Authors’ response:**

First, about the statistical analysis, in our original procedures we applied a binomial log (to estimate odds ratios) instead of a binomial log (to estimate prevalence ratios) and did not apply the binomial logit yet. Since we applied the binomial log in our original analysis we simply changed the phrase “odds ratio” into “prevalence ratio” in the manuscript as per your suggestion in the previous comment.

Regarding your suggestion about weights for survey analysis, we believe that we do not need to apply weighting in our survey analysis since we took equal samples from each village (“ganda” in the manuscript) as you can see in the “sampling procedure” section in the manuscript. We took equal proportion of the study samples from each village because of the difficulty posed by COVID-19 to enumerate the target population at that moment.

Thank for the idea about how other covariates could affect the associations. In our initial analysis we evaluated the association between the dependent variable and independent variables one by one. Then we conducted binomial logistic regression on stepwise forward (conditional) method to assess the adjusted associations using variables which showed unadjusted (individual) association on univariate regression analysis.

Finally we executed binomial logistic regression by entering the entire variables of the study using similar entry method to evaluate in case there is/are confounders which affect the observed associations and the model gave the same result as of the previous binomial logistic regression. This indicates that even if some variables did not included in our predictive analysis we evaluated and determined that they have no effect on the observed associations.

Related to your suggestion to evaluate the consistency of the term "predictor", we checked and fixed the consistency.

Furthermore, we added a limitation paragraph in the discussion section.

Writing typos checked and cleaned; N(%) added in Table 4 at the heading, and we changed the number "1" with "Ref." at table 5 to detail the category of reference.

7. PLOS authors have the option to publish the peer review history of their article ([what does this mean?](https://journals.plos.org/plosone/s/editorial-and-peer-review-process#loc-peer-review-history)). If published, this will include your full peer review and any attached files.


If you choose “no”, your identity will remain anonymous but your review may still be made public.


**Do you want your identity to be public for this peer review?** For information about this choice, including consent withdrawal, please see our [Privacy Policy](https://www.plos.org/privacy-policy).

Reviewer #1: No

- **Authors’ response:** Thank you and we respect the reviewers’ interest to remain anonymous in the option to publish the peer review history of our article.

[NOTE: If reviewer comments were submitted as an attachment file, they will be attached to this email and accessible via the submission site. Please log into your account, locate the manuscript record, and check for the action link "View Attachments". If this link does not appear, there are no attachment files.]

While revising your submission, please upload your figure files to the Preflight Analysis and Conversion Engine (PACE) digital diagnostic tool, <https://pacev2.apexcovantage.com/>. PACE helps ensure that figures meet PLOS requirements. To use PACE, you must first register as a user. Registration is free. Then, login and navigate to the UPLOAD tab, where you will find detailed instructions on how to use the tool. If you encounter any issues or have any questions when using PACE, please email PLOS at [figures@plos.org](mailto:figures@plos.org). Please note that Supporting Information files do not need this step.

*In compliance with data protection regulations, you may request that we remove your personal registration details at any time.* [*(Remove my information/details)*](https://www.editorialmanager.com/pone/login.asp?a=r)*. Please contact the publication office if you have any questions.*
